# All eyes on attention

**DOI:** 10.7554/eLife.77544

**Published:** 2022-03-15

**Authors:** Alessandro Benedetto, Martina Poletti

**Affiliations:** 1 Department of Brain and Cognitive Sciences and Center for Visual Science, University of Rochester New York United States; 2 Department of Brain and Cognitive Sciences, the Center for Visual Science and the Department of Neuroscience, University of Rochester Rochester United States

**Keywords:** attention, microsaccades, superior colliculus, oculomotor system, visual suppression, neuromodulation, Rhesus macaque

## Abstract

Eye movements are neither necessary nor sufficient to account for the neural effects associated with covert attention.

**Related research article** Yu G, Herman JP, Katz LN, Krauzlis RJ. 2022. Microsaccades as a marker not a cause for attention-related modulation. *eLife* **11**:e74168. doi: 10.7554/eLife.74168.

You are a football player, running at full speed. The ball is at your feet, your gaze and ‘overt’ attention fixed on it. Suddenly, in the corner of your eye, a player from the opposing team appears. You do not move your eyes away from the ball, but your attention shifts to monitoring your surroundings and ensuring the opponent does not get in your way. This ‘covert’ attention allows the brain to keep track of the player without looking at them (Figure 1A).

**Figure 1.**
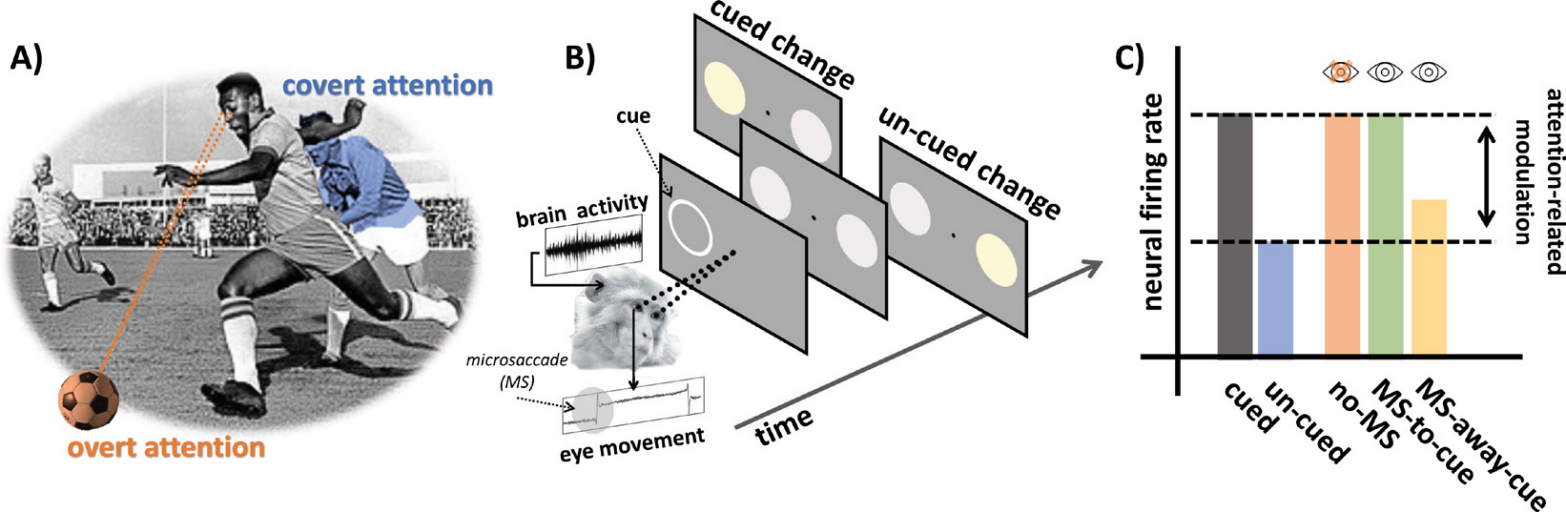
Examining the role of microsaccades in spatial attention. (**A**) An example of overt and covert attention: the player is looking at the ball (overt attention, in orange) and covertly paying attention to an approaching player from the opposing team (covert attention, in blue). (**B**) In the covert spatial attention task used by Yu et al., the monkey (which is holding a joystick) fixates on a dot in the middle of a screen. A brief visual cue is then flashed on the left or right of the screen (represented by a white ring), and two visual stimuli (disks) appear over the cued and un-cued location: one of these two stimuli then changes color. The monkey is instructed to release the joystick if the change appears at the cued location, and to hold the response otherwise. Yu et al. simultaneously recorded eye movements and brain activity from the superior colliculus. (**C**) Neural activity in the superior colliculus was enhanced for stimuli presented at the cued (gray) vs. un-cued (blue) location. The attention-related modulation was evident both for trials with microsaccades (MS) directed towards the cue (green; MS-to-cue), and for trials without microsaccades (orange; no-MS). The effect was substantially reduced when microsaccades were directed away from the cue (yellow; MS-away-cue).

Image credit: Alessandro Benedetto and Martina Poletti (CC BY 4.0); Monkey image adapted from Timothy Gonsalves (CC BY 4.0); Footballer image adapted from AFP/SCANPIX (Public domain).

According to the premotor theory of attention, when you were covertly monitoring the player, your brain was getting ready for (but not necessarily executing) eye movements towards that spot: this motor preparation would be both necessary and sufficient for attention to shift towards the new spatial location. In other words, spatial attention and motor preparation would share the same neural underpinnings. And indeed, the neural mechanisms and brain areas involved in the control of the fast eye movements (or saccades) needed to explore a scene are similar to those related to shifts in attention (Goldberg and Wurtz, 1972). In fact, activity in these areas is causally linked with how well individuals perform during attentional tasks (Cavanaugh and Wurtz, 2004).

Yet, recently, several studies have seriously challenged the assumptions that underlie the premotor theory of attention (see Smith and Schenk, 2012, for a review). In particular, they have demonstrated that attention can be shifted to different sites without preparing eye movements directed at those locations (Juan et al., 2004). In addition, different neural mechanisms mediate (or ‘modulate’) the changes in neural activity that are linked to covert spatial attention and the preparation for a shift in gaze (Li et al., 2021).

Still, new evidence has revived the idea that spatial attention may be intrinsically linked to eye movements. Even as you were keeping your gaze on the ball, your eyes were never stationary: they constantly moved due to tiny movements, or microsaccades, which shift the center of the gaze around the fixated location (for a review of this topic, see Rucci and Poletti, 2015).

Most likely, these microsaccades were directed towards the player coming your way – indeed, we tend to perform microsaccades towards covertly attended locations as we fixate on a different object. If these small eye movements are absent or directed away from the location which requires covert attention, neural and behavioral changes associated with attention disappear or decrease (Hafed and Clark, 2002; Lowet et al., 2018). This poses a serious problem to scientists. If generating microsaccades causes modulation of neuronal activity, the very idea of covert attention – where attention shifts without moving the eyes – may no longer be valid. Put differently, if small eye movements are causally linked to a change in attention, is there still room for the very concept of covert attention in our neuroscience handbooks?

Now, in eLife, Gongchen Yu, Richard Krauzlis and colleagues at the National Eye Institute, Bethesda and the University of Pittsburgh report cleverly designed experiments that help to address this crucial question (Yu et al., 2022). In particular, they managed to disentangle confounding factors which limited previous investigations into this topic (Meyberg et al., 2017).

The team recorded eye movement and activity in the superior colliculus (a brain area which integrates visual and motor information to initiate eye movements) in two monkeys trained to perform a covert spatial attention task (Figure 1B). The animals were required to fixate on a dot displayed on a monitor while holding a joystick. A brief visual cue was then flashed on the left or the right, automatically attracting the (covert) attention of the monkeys. Soon after, two visual stimuli appeared over the cued and un-cued location. One of these signals would then change color, and the monkeys were trained to release the joystick only if this switch took place at the cued location.

The results replicated well-known neural and behavioral attention-related effects: activity in the superior colliculus was enhanced when the visual stimulation occurred at the cued location (Figure 1C), and this increase correlated with monkeys being less likely to make mistakes during the task. Yu et al. then compared how neural activity modulation linked to attention differed when microsaccades were absent, directed towards the cued location, or away from it. Dissecting the relative contributions of microsaccades and spatial attention in this way revealed that neural modulation was present irrespective of microsaccades. In fact, it followed a very similar pattern of activity when microsaccades were absent or directed towards the cue (Figure 1C). Taken together, these findings demonstrate that microsaccades are not necessary for attention-related modulation in the superior colliculus.

The results provided by Yu et al. nicely complement previous behavioral studies which suggest that spatial attention can occur in the absence of microsaccades (Li et al., 2021; Meyberg et al., 2017; Poletti et al., 2017). Yet, outside of the lab, we rarely stare at dots on a screen the way test subjects are asked to do. In fact, in ‘real life’, microsaccades are often leveraged to precisely enhance fine spatial vision, and to explore the rich visual details which form the stimuli we hold at the center of our gaze, such as a fast-moving ball (Intoy and Rucci, 2020). Would the neural modulations associated with microsaccades reported by Yu et al. still occur in these more ecological settings? Only further research will be able to tell. In the meantime, this work makes a case for covert attention to remain in our neuroscience handbooks – for now.
